# Near-Infrared Fluorescence with Indocyanine Green to Assess Bone Perfusion: A Systematic Review

**DOI:** 10.3390/life12020154

**Published:** 2022-01-21

**Authors:** Marlies Michi, Max Madu, Henri A. H. Winters, Daniel M. de Bruin, Joost R. van der Vorst, Caroline Driessen

**Affiliations:** 1 Department of Surgery, Alrijne Hospital, Simon Smitweg 1, 2353 GA Leiderdorp, The Netherlands; 2 Department of Plastic Surgery, Amsterdam University Medical Center, Meibergdreef 9, 1105 AZ Amsterdam, The Netherlands; m.f.madu@amsterdamumc.nl (M.M.); h.winters@amsterdamumc.nl (H.A.H.W.); c.driessen@amsterdamumc.nl (C.D.); 3 Department of Biomedical Engineering and Physics, Amsterdam University Medical Center, Meibergdreef 9, 1105 AZ Amsterdam, The Netherlands; d.m.debruin@amsterdamumc.nl; 4 Department of Urology, Amsterdam University Medical Center, Meibergdreef 9, 1105 AZ Amsterdam, The Netherlands; 5 Department of Surgery, Leiden University Medical Center, Albinusdreef 2, 2333 ZA Leiden, The Netherlands; j.r.van_der_vorst@lumc.nl

**Keywords:** bone perfusion, near-infrared fluorescence, indocyanine green

## Abstract

*Background:* Adequate perfusion of a bone flap is essential for successful reconstruction of osseous defects. Unfortunately, complications related to inadequate bone perfusion are common. Near-infrared fluorescence (NIRF) imaging enables intraoperative visualization of perfusion. NIRF has been investigated in reconstructive surgery to aid the surgeon in clinical perioperative assessment of soft tissue perfusion. However, little is known on the beneficial use of NIRF to assess bone perfusion. Therefore, the aim of this review was to search for studies evaluating NIRF to assess bone perfusion. *Methods:* A systematic review, according to the Preferred Reporting Items for Systematic Reviews and Meta-Analyses (PRISMA) guideline, was performed. Studies up to October 2021 were included. We extracted data regarding the study population, size and design, reported objective fluorescence parameters and the methodology used for fluorescence imaging and processing. *Results:* Ten articles were included. Studies reported unevenly on the protocol used for NIRF imaging. Five studies reported objective parameters. Absolute and relative perfusion parameters and parameters derived from maximum fluorescence were reported. The clinical significance of these parameters has not been evaluated in humans. *Conclusion:* The evidence on bone perfusion as measured with NIRF is limited. More clinical studies are required.

## 1. Introduction

**Figure 1:**
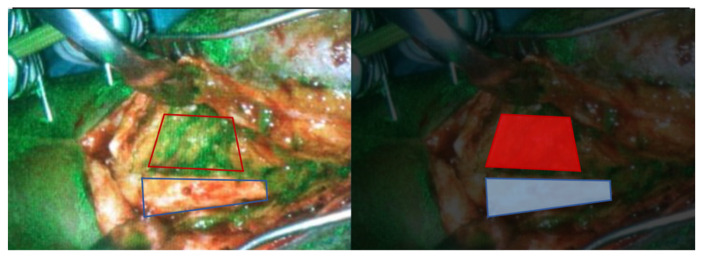
Perioperative photo of a patient who has a viable rectus femoris muscle flap (red inset) in a tibia after debridement of osteomyelitis. Because of ongoing infection and a PET scan suspicious for necrotic bone another surgical debridement was performed. Fluorescence imaging confirmed a lack of signal at a specific tibia region (blue inset) which was removed.

Adequate perfusion of tissue is a prerequisite for successful reconstructive surgery. The gold standard for tissue perfusion assessment in reconstructive surgery is based on perioperative clinical judgment of traditional parameters (skin colour, capillary refill and turgor, bleeding at wound edges) [1,2]. Bone perfusion is particularly difficult to clinically assess, as soft tissue related parameters are missing. Subjective observations such as bleeding of the periosteum or from multiple drill holes and bone edges are used. These observations however may be influenced by previous surgery, trauma or infection [3,4]. Unfortunately, complications related to inadequate tissue perfusion are common, despite clinical perioperative perfusion assessment [5,6,7]. Several techniques have been developed over the years to support surgeons in assessing the adequacy of tissue perfusion [8]. The ideal technique would be able to predict tissue viability by providing objective perfusion parameters. Near-infrared fluorescence (NIRF) imaging is a technique that enables intraoperative visualization of anatomic structures and perfusion of tissue after the administration of the fluorescent tracer indocyanine green (ICG) [9] (Figure 1). ICG is a non-specific, FDA approved, fluorescent tracer that binds to plasma proteins and is considered safe [9]. ICG has fluorescent properties when excited with light in the near-infrared range and can be visualized with an NIRF camera. This imaging method is rapidly gaining popularity within different fields of surgery and has been investigated in reconstructive surgery, mostly in breast reconstruction, showing promising results [10,11,12,13]. The advantage of NIRF compared to other real-time optical imaging, such as optical coherence tomography (OCT), sidestream darkfield microscopy (SDF), and laser speckle contrast imaging (LSCI), is its wide-field view, non-contact and less time-consuming technique [14]. Little is known about the use of NIRF to assess bone perfusion. The aim of this study is to present a comprehensive review of the literature concerning the assessment of bone perfusion with NIRF. The focus is to report which NIRF imaging methodology was used and whether objective perfusion parameters are available to allow differentiating between viable and non-viable bone in future research.

## 2. Methods

A systematic review, according to the Preferred Reporting Items for Systematic Reviews and Meta-Analyses (PRISMA) guideline, was performed to assess current concepts regarding the perfusion assessment of bone with fluorescence imaging. A PubMed database search was conducted with the following search strategy:

(“indocyanine green”[MeSH Terms]) OR (“indocyanine green”[Title/Abstract]) OR (“ICG”[Title/Abstract]) OR (fluorescence imaging*[tiab]) OR (wofaverdin[tiab]) OR (vophaverdin[tiab]) OR (vofaverdin[tiab]) OR (cardio green[tiab]) OR (cardiogreen[tiab])) AND (“Bone and Bones”[Mesh] OR bone[tiab] OR bones[tiab].

References of the selected articles were screened to identify additional papers. The search is up to date to October 2021. Articles written in English and Dutch were included. Two independent reviewers (MMi and MM) screened through the abstracts and selected articles relevant to this review. In case of discordance, a third reviewer (CD) was consulted until consensus was reached. Inclusion criteria were human, cadaveric or animal studies with an evidence level ranging from 1 to 5, investigating bone perfusion by ICG (qualify and quantify). There was no minimum number of participants. In vitro studies were excluded. Methodological quality and risk of bias of the included case series were assessed using the “Joanna Briggs Institute” (JBI) checklist. Level of evidence of the studies was assessed by the American Society of Plastic Surgeons (ASPS) Evidence Rating Scale.

## 3. Data Collection

From the included articles, we extracted data regarding study population, size and design, study end points, reported objective fluorescence parameters and the methodology used for fluorescence imaging and processing (camera type, positioning, ICG dosage, software used for data analysis). To provide comparable results within studies, we contacted the authors to obtain additional information.

We performed a descriptive and qualitative analysis of the data extracted; no meta-analysis was possible due to the heterogenicity of the studies included.

## 4. Results

**Figure 2:**
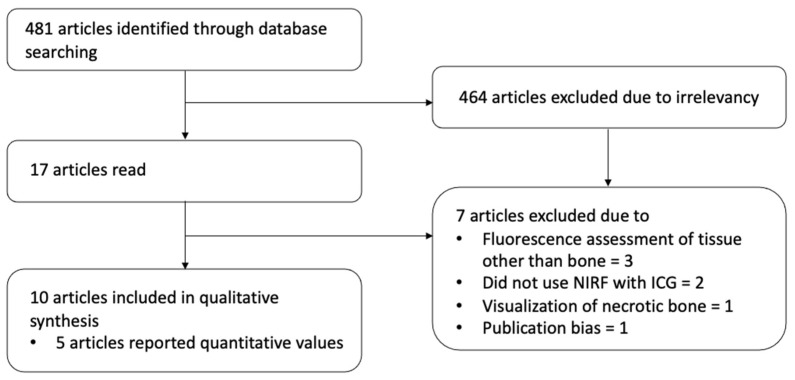
Flowchart of inclusion.

The initial search in PubMed identified 481 articles. Of these, 464 were excluded because they were not relevant according to title or abstract. 17 articles were fully read, 10 articles met the inclusion criteria and were included into the study (Figure 2). One article was excluded due to publication bias because the dataset was already included in another paper. Three articles were excluded because these focused on assessment of fluorescence of tissue other than bone (sarcoma, mucosa, flap pedicle), two articles were excluded because these used another method than NIRF with ICG to visualize bone (different probe for fluorescence). One article was excluded because it focused on visualization of necrotic bone.

The characteristics of the included studies are presented in Table 1. The first study to report the use of ICG to assess bone perfusion was in 2012 by Nguyen et al. [15]. The number of cases per study varied between 2 and 39. Three studies reported on the NIRF assessment of bone perfusion in animals, three on cadavers and four in human beings. Only one prospective study in humans was identified [16]. Perfusion assessment of bone cortex and/or medulla with NIRF was reported in tibia, fibula, radius, iliac crest, femur and scapula.

### 4.1. NIRF Imaging Methodology

Not all studies reported clearly on the protocol used for NIRF imaging. Six different imaging systems were used combined with differing NIRF imaging processing software suites (Table 2). Position of the camera from the surgical field was 30 cm in most cases. The intravenous ICG dose, when reported, varied from 0.036 to 0.3 mg/kg or 7.5 mg to 25 mg. The duration of NIRF imaging video recording to obtain an ICG fluorescence curve over time was reported in two studies only and was 3 to 4 min.

### 4.2. Objective Perfusion Parameters

Quantification of NIRF imaging was reported only in five studies [15,16,17,18,19] (Table 3). Three studies reported on absolute perfusion units, defined as maximum fluorescence intensity after ICG injection [16,17,19]. Additionally, relative perfusion units were reported, which is the fluorescence intensity of the region of interest in relation either to another region of the flap where perfusion is considered to be optimal or to background fluorescence [15]. Three studies calculated several additional perfusion parameters.

### 4.3. Maximum Bone Fluorescence Intensity

**Figure 3:**
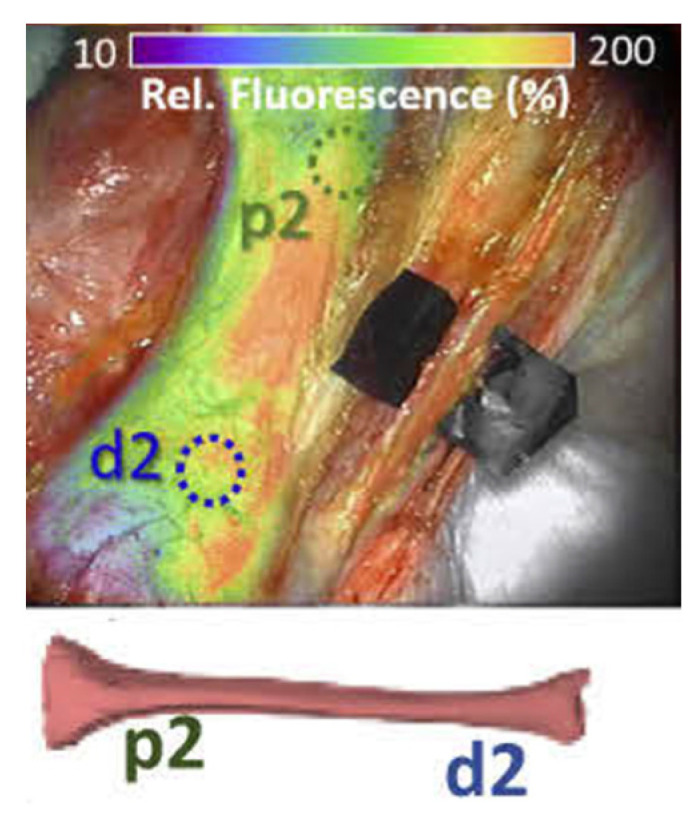
ICG fluorescence map overlay on the white light images of a porcine tibia from the study of Gitajn et al. p2 and d2 are, respectively the proximal and distal region of interest of the tibia. The fluorescence imaging had been enhanced for better visualization.

Fichter et al. found that the maximum bone marrow fluorescence intensity before and after osteotomy decreased, but not significantly [16]. Valerio et al. described in their study that all their (chimeric) osseous flaps had an absolute fluorescence value higher than 6 on a 0–255 grey scale system assigned by the SPY-Q software [17]. The value on this scale is based on the fluorescence intensity of the image, with a higher number corresponding with a higher fluorescence intensity. The lower threshold of 6 was derived from earlier studies in skin sparing mastectomy patients [20]. The exact absolute fluorescence value per osseous flap was not described. NIRF was used in guiding the resection of inadequately perfused tissue. No complications related to inadequate bone perfusion were reported. A special kinematic model was used by Gitajn et al. in twelve porcine tibias with progressive conditions mimicking progressive bone trauma of increasing severity (Figure 3) [19]. Although maximum fluorescence intensity was not significantly different between normal and injured bone, the proprietary bone-specific kinetic model was able to discriminate between normal and injured bone. This is reported further in paragraph “Other parameters”.

### 4.4. Relative Bone Fluorescence Intensity

Nguyen et al. described an intensity ratio (fluorescence at region of interest/background fluorescence) for quantification of fluorescence [15]. Vascularized bone marrow had a signal/background ratio of 2.30 and devascularized bone marrow, simulated by clamping the vascular pedicle, of 1.03.

### 4.5. Other Parameters

Two derived perfusion parameters were reported by Fichter et al., namely the wash in rate (WiR) (defined as the maximum fluorescence slope) and wash in perfusion index (WiPI) (defined as the ratio between the wash in area under the curve (WiAUC), defined within the start of rise of the fluorescence slope and the maximum fluorescence and rise time). Both parameters were significantly decreased before and after osteotomy [16]. No cut-off value of adequate perfusion was suggested as no correlation to clinical outcome was made.

Elliott et al. developed a kinetic analysis model to capture and describe the bipartite blood supply (periosteal and endosteal) to cortical bone [18]. They applied this kinetic model to the ICG fluorescence curves of regions of interest in bone to described recovered values for endosteal (late) and periosteal (early) blood supply to the bone. With the use of this model by Gitajn et al., recovered values of early, late and total blood perfusion (EBP, LBP, TBP) and late perfusion fraction (LPF) significantly differed between injured and normal bone [19]. A combination of LBP and LPF had a 90% sensitivity, 88% specificity and 89% accuracy in diagnosing injured bone.

## 5. Discussion

This systematic review reports on studies that have explored the role of NIRF imaging with ICG in assessing bone perfusion. The studies evaluated in this review show that it is a promising way of visualizing bone perfusion, although there is a clear lack of data on this technique.

The goal of real-time bone perfusion imaging in a clinical setting is primarily to differentiate between viable and non-viable tissue, to allow image-guided tailoring of osseous flaps or debridement of necrotic bone. An ideal imaging technique should allow clear differentiation between viable and non-viable tissue, be easy to set up, easy to use and interpret, be safe and minimally invasive for patients, provide consistent results between cases and ideally be cost-effective. Objective perfusion parameters could allow such differentiation, with a clear cut-off value indicating which tissue is deemed non-viable. While objective perfusion parameters derived from NIRF imaging have been reported previously for soft tissue, the evidence for objective levels of bone perfusion is scarce [13]. There is no consensus on which parameters should be used to ideally assess bone perfusion.

In cadaveric studies the feasibility of ICG to penetrate cancellous (endosteal) portion of bone through penetrating periosteal capillaries has been shown [21,22,23]. NIRF with ICG should therefore allow adequate assessment of bone perfusion. NIRF with ICG has been used successfully to subjectively assess bone perfusion in two retrospective case studies [24,25]. Yoshimatsu et al. (2017 and 2019) even suggested that ICG could be used to define bone specific perfusion regions or bone angiosomes in new osseous flaps when injected into specific arteries. However, cadaveric studies that evaluate bone perfusion are limited and may over- or underestimate the vascular territory of bone flaps as the dynamic aspect of perfusion is not considered.

Five studies reported objective outcomes, including absolute, relative and extrapolated perfusion parameters. Some papers reported human studies and others animal research. Additionally, the NIRF imaging methodology used showed high heterogeneity between the studies included. For example, the studies used different camera systems with different camera chips and optics, resulting in varying camera sensitivities to pick up fluorescence. Moreover, several camera systems employ intensity optimization algorithms to boost the captured image data. Keeping these factors mind, there is a challenge to compare cut-off values between studies using different systems.

Absolute perfusion values, defined as maximum fluorescence in number of units after ICG injection at a certain time point, were reported by Fichter et al. (2019), Valerio et al. (2015) and Gitajn et al. (2020). While Fichter et al. (2019) and Gitajn et al. (2020) looked at maximum fluorescence on a fluorescence perfusion curve over time, Valerio et al. (2015) looked at maximum fluorescence at a single not further specified time point. Fichter et al. (2019) looked at cancellous bone in a transverse view of a fibula flap and stated that maximum fluorescence did not differ significantly before and after osteotomy. No correlation to clinical outcome was made as they stated that the impact of decrease in bone perfusion, and consequently variation in perfusion parameters, on bone healing is difficult to assess. Biomechanical forces, wound bed configuration, adequacy of osteosynthesis, postoperative infections might all influence bone healing. Gitajn et al. (2020) found no significant difference in absolute perfusion between injured and healthy porcine bone. Valerio et al. (2015) used an absolute minimal fluorescence value of 6 as a cut off value for acceptable bone perfusion based on earlier studies in skin sparing mastectomy patients [20].

Nguyen et al. (2012) reported a relative perfusion value; the fluorescence signal (fluorescence at region of interest) was divided by the background fluorescence. Vascularized bone marrow had a signal/background ratio of 2.30 and devascularized bone marrow of 1.03. This study therefore showed that avascular bone has a fluorescence signal almost equivalent to background fluorescence. The question remains how progressive injury of bone influences this ratio.

Finally, the research groups of Gitajn et al. (2020) and Elliot et al. (2019), proposed and applied a new kinetic model to ICG fluorescence curves of regions of interest in bone under several injury conditions. The underlying concept of this model is that bone ICG fluorescence curve represents a combination of “late” and “early” bone perfusion, which in turn, reflects the bipartite blood supply network of bone. Bone has a bipartite blood supply, based on a periosteal and an endosteal network [26,27,28]. These networks are connected through small capillaries. This intricate network allows sustained bone perfusion even when the nutrient artery providing the endosteal perfusion is lost, injured or not included in a bone flap [23,28]. With this model Gitajn et al. (2020) and Elliot et al. (2019) extrapolated several parameters and concluded that a combination of LBP and LPF values had a 90% sensitivity, 88% specificity and 89% accuracy in diagnosing injured porcine bone. However, it is difficult to extrapolate their methods and results because others have not used this model yet.

Recent studies suggest that NIRF with ICG in breast reconstruction is cost effective [29]. We did not find any cost-analysis studies on the use of NIRF to assess bone perfusion.

Future studies on NIRF assessment of bone perfusion should report objective outcome measures, including maximum and relative bone fluorescence intensity, after their use is confirmed in fundamental studies. When reporting absolute perfusion parameters, van den Hoven et al. propagated the normalization of the time-intensity curves to minimize the effect of multiple influencing factors, including camera distance and ICG dosage [30]. Bipartite bone perfusion raises the question on ideal NIRF camera position in relation to the evaluated bone. When the fluorescence camera is oriented perpendicular to a transverse bone cut, initially separate fluorescence images of periosteal, cortical and medullary bone could be observed. When the bone is viewed in a sagittal direction, fluorescence from periosteal, cortical and medullary bone will overlap and provide a combined view. The best way to assess bone, and whether there is a clinically relevant difference in evaluation remains unknown. Based on the available data, a camera position of 30 cm, perpendicular to the region of interest, and a dose of 0.1 mg/kg, followed by flushing with saline would be recommended [13]. NIRF depth assessment is dependent on the tissue optical density which is responsible for the absorption and scattering of light. Studies report on the depth of NIRF assessment of soft tissue (liver, skin and fat) being 3 to 15 mm [31,32]. We found no studies evaluating the depth of penetration of NIRF in bone tissue. It is thus unknown to what extent NIRF assessment is reliable in bone tissue. Nevertheless, a transverse cut of bone will be able to both assess periosteal, cortical and medullary bone perfusion. NIRF is able to record fluorescence inflow and outflow [33]. As perfusion is a dynamic process, recording should be conducted for at least 180 s to be able to record a perfusion (fluorescence) curve over time that includes inflow and outflow. To assess venous congestion, which is considered to be a great problem in reconstructive surgery, it could be considered to prolong the recording up to, for example, ten minutes. External parameters should also be included in analyses (temperature, systemic medication during the operation). Finally, it is also recommended to calibrate the system intensity response when several systems are used within a study.

It is a challenge to implement the NIRF findings of bone perfusion in surgery. Regarding reconstructive surgery, NIRF assessment of the bone flap after osteotomy, drilling and plating, e.g., in a case of mandibular reconstruction, will often not lead to an adjustment of the flap itself. However, consequent data of NIRF and clinical follow up, may eventually be useful in assessing the maximal length/surface and number of osteotomies of a bone flap. The more NIRF data available on healthy bone perfusion, the better we will be at assessing inadequate perfusion due to infection, trauma or radiotherapy. Ultimately, it may facilitate in guiding the debridement of an acceptor site before reconstruction.

One of the limitations of this systematic review was the limited amount and heterogeneity of studies included. Therefore, it was not feasible to perform a meta-analysis.

## 6. Conclusions

Based on the results of this review, the evidence on NIRF assessment of bone perfusion is limited but promising. A few objective parameters have been reported, but their clinical significance has not been evaluated in humans. All the reported objective values are not yet available in real-time, limiting their applicability during surgery. More studies are required to assess clinical significance and cost effectiveness of NIRF with ICG to differentiate between viable and non-viable tissue, to allow image-guided tailoring of osseous flaps or debridement of necrotic bone.

## Figures and Tables

**Table 1:** Included studies.

	Study	Level of Evidence	Study Design	Total Number of Cases	Reported Objective NIRF Values	Non-Viable Tissue Excised
1	Nguyen, 2012	5	Experimental research (animal)	8 animals/16 limbs	Yes	No
2	Valerio, 2015	5	Retrospective case series	16	Yes	No
3	Yoshimatsu, 2017	5	Experimental research (cadavers)	4 cadavers/8 limbs	No	No
4	Tyrell, 2018	5	Retrospective case series	2	No	Yes
5	Fichter, 2019	2	Prospective case series	39	Yes	No
6	Yoshimatsu, 2019	5	Experimental research (cadavers)	8 cadavers	No	No
7	Elliot, 2019	5	Experimental research (animal)	2 animals	Yes	No
8	Gitajn, 2020	5	Experimental research (animal)	12 animals	Yes	No
9	Muangsiri, 2021	5	Experimental research (cadavers)	11 cadavers/22 flaps	No	No
10	Reece, 2021	5	Retrospective case series	14	No	No

**Table 2:** Fluorescence imaging methodology.

	Study	ICG Dose	ICG Fluorescence System	Software	Image Timeframe	Camera Position	Area of Interest
1	Nguyen, 2012	36 ug/kg IV	FLARE imaging system	Custom	NR	18 inches from surgical field	1. Radius of forelimb 2. Distal osteotomy site of fibula
2	Valerio, 2015	7.5 mg IV	SPY and SPY Elite	SPY-Q	NR	Laser assisted	Periosteum and cancellous bone of several flaps
3	Yoshimatsu, 2017	NR (5 mL Pulsion ICG solution)	Sony HD, Handycam CM 05	NR	NR	NR	Periosteum and cancellous bone of the iliac crest bone flap
4	Tyrell, 2018	NR	SPY	NR	NR	NR	Sternum
5	Fichter, 2019	0.3 mg/kg IV	Pulsion Photodynamic Eye	ImageJ and Prism	3 min	30 cm	Distal osteotomy line of distal fibular segment
6	Yoshimatsu, 2019		Sony HD, Handycam CM 05	NR	NR	NR	Periosteum and cancellous bone of medial condyle of femur bone flap
7	Elliot, 2019	NR	Zeiss Pentero OPMI 800 in FLOW 800 mode	MatLab	NR	30 cm	Tibia, at baseline, after osteotomy with intact and disrupted periosteum, proximal or distal
8	Gitajn, 2020	0.1 mg/kg	Zeiss Pentero OPMI 800 in FLOW 800 mode	MatLab	4 min	30 cm	Tibia, at baseline, after osteotomy with intact and disrupted periosteum, proximal or distal
9	Muangsiri, 2021	25 mg (Daiichi Sankyo, Diagnogreen)	Fluoptics	NR	NR	NR	Periosteum, cortex and cancellous bone of clavicula flaps
10	Reece, 2021	NR	SPY elite	NR	NR	NR	Visualization of bone surface of ilac crest bone flap

**Table 3:** Reported objective parameters.

	Study	ICG Fluorescence System	Perfusion Parameters
1	Nguyen, 2012	Flare	Absolute perfusion: NR
			Relative perfusion: Fluorescence intensity of the region of interest/Fluorescence Intensity of background · Vascularized bone marrow and devascularized bone marrow showed a signal/background ratio of 2.30 and 1.03, respectively
			Other parameters: NR
2	Valerio, 2015	SPY and SPY Elite	Absolute perfusion: SPY-Q score greater than 6.0
			Relative perfusion: NR
			Other parameters: NR
5	Fichter, 2019	Pulsion Photodynamic Eye	Absolute perfusion: Peak Enhancement (PE) · Before osteotomy: 246 (140–255) · After osteotomy: 244 (111–255) No significant difference
			Relative perfusion: NR
			Other parameters: Wash in rate (WiR): · Before osteotomy 6.42 (2.26–38.46) · After osteotomy 4.41 (0.20–51.14) Significant difference (0.034) Wash in perfusion index (WiPI) · Before osteotomy 114.2 (48.4–188.3) · After osteotomy 84.4 (29.0–197.5) Significant difference (0.0179) Other reported parameters Ti—Time local TTP—Time to peak WiAUC—Wash in area under the curve RT—Raise time
7	Elliot, 2019	Zeiss Pentero	Absolute perfusion: NR
			Relative perfusion: NR
			Other parameters: PBF (periosteal blood flow): Baseline 6.8 ± 1.1 mL/min/100 gr After periosteal damage PBF ROI adjacent to periosteal damage decreased by 21.8 ± 22.3% PBF ROI at site of periosteal damage decreased by 83.8 ± 4% EBF (endosteal blood flow) constant between 0.9 and 1.4 mL/min/100 gr After transection of bone in two locations and periosteal damage PBF decreased by 52.5 ± 17.6% EBF decreased by 73.5 ± 6.0% Additional calculated parameters: Total Blood Flow (TBF) = PBF + EBF EBF fractional flow (EFF) = EBF/TBF
8	Gitajn, 2020	Zeiss Pentero en Spy Elite	Absolute perfusion: Imax (a.u) Normal bone 201 ± 50 Injured bone 163 ± 58 No significant difference
			Relative perfusion: NR
			Other parameters: Total bone perfusion (TBP) normal vs. injured bone: 3.2 ± 0.8 vs. 0.8 ± 0.4 mL/min/100 gr Early bone perfusion (EBP) normal vs. injured bone: 3.0 ± 0.8 vs. 0.7 ± 0.4 mL/min/100 gr Late bone perfusion (LBP) normal vs. injured bone: 0.1 ± 0.0 vs. 0.2 ± 0.1 mL/min/100 gr Late perfusion fraction (LPF) normal vs. injured bone: 2.5 ± 1.1 vs. 21.4 ± 10
